# Nurses’ perceptions of family-centred care in neonatal intensive care units: Rapid qualitative evidence synthesis of studies in low- and middle-income countries

**DOI:** 10.4102/hsag.v31i0.3236

**Published:** 2026-02-11

**Authors:** Clara Wepener, Elrietha Olivier, Berna Gerber

**Affiliations:** 1 Department of Health and Rehabilitation Sciences, Division Speech-Language & Hearing Therapy, Faculty of Medicine and Health Sciences, Stellenbosch University, Cape Town, South Africa

**Keywords:** family-centred care, FCC, neonatal care, NICU, nurse perceptions, qualitative evidence synthesis

## Abstract

**Background:**

Family involvement in the neonatal intensive care unit (NICU) benefits both the infant and the family; however, nurses working in the NICU in low- and middle-income countries (LMICs) face unique contextual challenges that influence the practice of family-centred care (FCC).

**Aim:**

This rapid review aims to synthesise nurses’ perceptions of FCC in NICUs in LMICs and to identify factors influencing its implementation in these contexts.

**Method:**

Electronic databases were searched for relevant studies published between 2020 and 2025. A Preferred reporting items for systematic reviews and meta-analyses (PRISMA) diagram depicts the study selection process. The Joanna Briggs Institute’s (JBI) Critical Appraisal Checklist for Qualitative Research was used for quality assessment of the selected articles, and data were charted using the JBI extraction instrument. Descriptive thematic synthesis was used to synthesise and report the findings.

**Results:**

The search generated 1598 articles, with 9 studies from 5 different LMICs meeting the selection criteria. The synthesis of the findings resulted in the identification of 6 themes, namely policies and resources, cultural and contextual variables, nurses’ roles and responsibilities, nurse-family dynamics, staff dynamics and support and training.

**Conclusion:**

Nurses in LMICs expressed varied views on involving families in NICUs and highlighted the influence of cultural and contextual factors. They emphasised the need for clear guidelines, adequate resources and appropriate support and training to enable successful implementation.

**Contribution:**

The review provides a single point of access of contextualised synthesised findings of factors influencing the implementation of FCC in LMIC NICUs.

## Introduction

Approximately, 15 million babies are born prematurely annually. Premature babies have a lower survival rate, especially in low-income settings and a greater risk of disability, especially in middle-income settings (World Health Organization [WHO] 2023). To counter these risks and to improve the care of premature and low birth-weight infants, the WHO Department of Maternal, Newborn, Child and Adolescent Health and Ageing launched a new set of 25 recommendations in 2022. The document aims to provide individuals involved in the care of premature infants with guidelines that include recommendations on care for complications, preventive and promotive care and family involvement and support (WHO 2022).

In neonatal intensive care units (NICUs), nurses have traditionally fulfilled essential responsibilities in preventive and promotive care, as well as managing medical complications (Foster et al. 2024). Over the past six decades, increased recognition of the importance of family involvement and support in shaping neonatal outcomes has led NICUs in numerous countries to adopt a family-centred care (FCC) approach, with nurses playing the most prominent role in its implementation (Reid, Bredemeyer & Chiarella 2021). Family-centred care aims to: (1) treat the patient and family with respect and dignity; (2) share timely, complete and accurate information; (3) encourage and support participation in the care and decision-making at the level that families choose and (4) ensure collaboration between the role players (Institute for Patient- and Family-Centered Care n.d.).

Recent reviews unpack the concept of FCC in NICUs in more detail. A review by Abukari and Schmollgruber (2023) discusses 10 subthemes under the four key FCC concepts, highlighting aspects such as considering the family needs and socio-economic backgrounds, building rapport, educating families and providing easily understood information, involving and supporting families, the need for collaboration in terms of facility design and resources and professional training and policy implementation. A concept analysis with similar findings by Larocque et al. (2021) emphasises that one of the attributes of FCC is a genuine partnership between the families and the healthcare practitioners. However, these reviews are synthesised mainly from studies originating from high-income countries (HICs), and studies exploring the concepts of family partnership and involvement in decision-making, medical rounds and interdisciplinary family conferences are lacking in Africa and Asia (Vetcho, Cook & Ullman 2020). It is important to observe that the extent to which families are involved in the NICU differs across contexts (Lazzerini et al. 2024) and that although the concept of FCC is well recognised in literature, it is not yet fully accepted in many NICUs across the world (Ding et al. 2019).

A partnership between families and healthcare practitioners is not always easy. The family of the hospitalised infant (as well as the infant) may experience high levels of stress in the hospital setting (Mallapu et al. 2023). Factors such as concerns about their infant’s medical status, the family’s socio-economic situation, home and work responsibilities and their own health concerns can play a role in the family’s involvement in the care of the infant in the NICU (Soni & Tscherning 2021).

From a nursing perspective, balancing all the neonatal nursing roles in a NICU is reported to be rewarding but also stressful and demanding (Fiske 2018). Family-centred care advocates for the families of the hospitalised infants to become part of the team, possibly placing additional personal, cultural and contextual challenges on the nursing staff who may be dealing with concerns such as staffing issues, teamwork difficulties and challenges in their physical environment (Bry & Wigert 2022; Soni & Tscherning 2021). However, when families are involved in the care of their hospitalised infants, it benefits both the parents and the infant and enables earlier hospital discharge of the infant (Gómez-Cantarino et al. 2020). It is thus important that the attitudes and the mindsets of healthcare practitioners who need to implement this type of care be considered for successful implementation (Gómez-Cantarino et al. 2020; Oude Maatman et al. 2020).

Chan and Shorey (2021) reviewed and consolidated healthcare practitioners’ perspectives on parental participation in the NICU. Their findings show that although many think family involvement is beneficial for infants and families, many challenges are also reported. These include inconsistent guidelines and policies, a lack of leadership, uncertainty about roles and responsibilities, unpredictable and heavy workloads with staff shortages and poor work organisation, challenges communicating with parents as well as other staff members, frustrations training and involving certain parents, the layout of the environment, uncertainty around capabilities to provide parental support and the emotional strain of doing so (Chan & Shorey 2021).

According to Bronfenbrenner’s bioecological model, a child’s development is influenced by a complex system of interactions, particularly in their immediate environmental context (Bronfenbrenner & Evans 2000). In the NICU, the infant’s interactions differ vastly from those in the home environment. Although many challenges related to family involvement in NICUs are universal, it is important to consider context-specific factors, particularly in low- and middle-income countries (LMICs), where the negative impact of prematurity is most pronounced. While understanding that the perspectives of all stakeholders involved in FCC are important (Kutahyalioglu et al. 2023), this review focusses on nurses, given their central role in its implementation. Building on previous reviews, the rapid synthesis of the perception of the nurses involved in FCC in LMIC hospital settings is one step towards informing best practice of this approach in these settings.

### Objectives

The objectives for the review were:

- to synthesise recent qualitative research evidence on nurses’ perceptions regarding FCC in NICUs in LMI settings
- to identify and explore factors influencing the implementation of FCC in NICUs in LMI settings

## Research methods and design

The review followed the guidelines for rapid qualitative evidence synthesis (QES) outlined by Booth et al. (2024) as well as consulting the Cochrane protocol for QES (Glenton et al. 2023). Adding to previous reviews on the topic of FCC in the NICU (e.g. Abukari & Schmollgruber 2023; Chan & Shorey 2021; Ding et al. 2019; Vetcho et al. 2020), a rapid synthesis of recent evidence with the specific focus on nurses’ perceptions in LMICs provides a single point of access for contextualised synthesised evidence (Langlois et al. 2019).

### Criteria for inclusion and exclusion

Rapid QES studies have well-defined criteria to ensure that the search is focused and provides the needed evidence (Booth et al. 2024). The gross national income (GNI) per capita of a country (LMIC versus HIC) was not included in the search criteria but was used as a criterion in the study selection. Although it is problematic to draw distinctions according to these criteria (Khan et al. 2022), it is performed in an attempt to hear the voices that are less represented in the literature and that are more relevant to the reviewers’ context.

#### Types of studies

Primary studies with pure qualitative designs and presenting direct evidence were included in the search. Existing reviews such as scoping, systematic or literature reviews identified during the search were integrated into the background of this review.

#### Participants

Studies with nurses as participants were considered for inclusion. Studies including nurses and other healthcare practitioners and/or parents were also considered for inclusion, as the number of LMIC studies was limited. Only data pertaining to nurses’ perceptions and experiences were extracted from the selected studies.

#### Concept

The primary focus of this review was on the perceptions and experiences of nurses providing FCC in the NICU. Perceptions specific to the impact of the coronavirus disease 2019 (COVID-19) pandemic or palliative neonatal care were not included. Studies in LMIC referring to family involvement without using the terminology FCC were considered for inclusion.

#### Context

Studies performed in LMICs where nursing was delivered in a neonatal hospital setting including NICUs and neonatal units providing similar specialised care were considered for inclusion. The World Bank Classification was used to determine whether a country was classified as an LMIC or HIC (https://datahelpdesk.worldbank.org/knowledgebase/articles/906519-world-bank-country-and-lending-groups).

### Search methods for identification of studies

The databases MEDLINE via PubMed, CINAHL, Scopus and Health Source: Nursing/Academic Edition were used to identify relevant studies, in consultation with a health librarian. As the review was rapid, a limited number of relevant databases were searched for English peer-reviewed articles published between 2020 and 2025. The search string used was: ‘nurse or nurses or nursing or healthcare professional or health personnel AND perception* or experience* or view* or attitude* or belief* or understanding or perspective* or opinion* AND family centered care or family centered nursing or FCC or family-centred nursing or family or parent AND NICU or neonatal intensive care unit or special care or baby unit or newborn intensive care’.

### Study screening and selection

All studies identified through the database searches were transferred to the web-based application Rayyan to assist with the removal of duplicates and enable an efficient screening process (Ouzzani et al. 2016). After the duplicates were removed, two reviewers in the review team first screened 15% of the records on Rayyan based on the title and abstract to ensure that consistency was achieved according to the criteria outlined above. The primary reviewer screened the remaining records and resolved uncertainties with the second and third reviewers. The primary reviewer then conducted a full-text screening of the selected records, focusing on privileging diverse and rich qualitative studies (Booth et al. 2024; Glenton et al. 2023). The second reviewer screened 20% of the full-text articles, and the third reviewer was consulted to resolve any uncertainties.

### Quality assessment

The JBI Critical Appraisal Checklist for Qualitative Research was used to ensure that the reviewers reflected on the strengths and limitations of the considered studies (Lockwood, Munn & Porritt 2015). The primary reviewer used this checklist to assess whether the selected articles addressed its items, including congruency between philosophical perspectives and methodology, methodology and objectives, steps within the methodology, methodology and results and interpretation, ethics approval, audibility of the voices of the participants, as well as the self-reflection of the researcher and their interaction with the research.

### Data extraction

The JBI Data Extraction Tool for Qualitative Research was used as a reference to extract relevant data from the selected studies (Lockwood et al. 2015). Data extraction was conducted by the primary reviewer and involved systematically recording the following information in a structured table: author and year of publication, the methodology and methods, the phenomenon of interest, the population (including sample size), the setting and context (including geographical and cultural information), the outcomes, as well as recording relevant quotations from the included studies.

### Evidence analysis and synthesis

Descriptive thematic synthesis was used to synthesise and interpret the data from the included studies using first-order constructs (participants’ accounts) and second-order constructs (authors’ interpretations) (Thomas & Harden 2008). Mainly first-order constructs were analysed to ensure that the nurses’ perspectives could be separated from the perspectives of other participants. Second-order constructs were included when they specifically refer to nurses’ perspectives.

The qualitative data analysis software ATLAS.ti 24 was used to assist in data management and analysis. The analysis followed the steps outlined by Braun and Clarke (2006). The primary reviewer familiarised themselves with the data by reading and re-reading the uploaded transcripts. Initial codes were then generated using line-by-line coding and a cyclical process of refinement. Codes with similar meanings were subsequently grouped together to form themes. These themes were reviewed and redefined around key concepts that emerged inductively from the data.

### Ethical considerations

This article followed all ethical standards for research without direct contact with human or animal subjects.

## Results

### Search results

**FIGURE 1:**
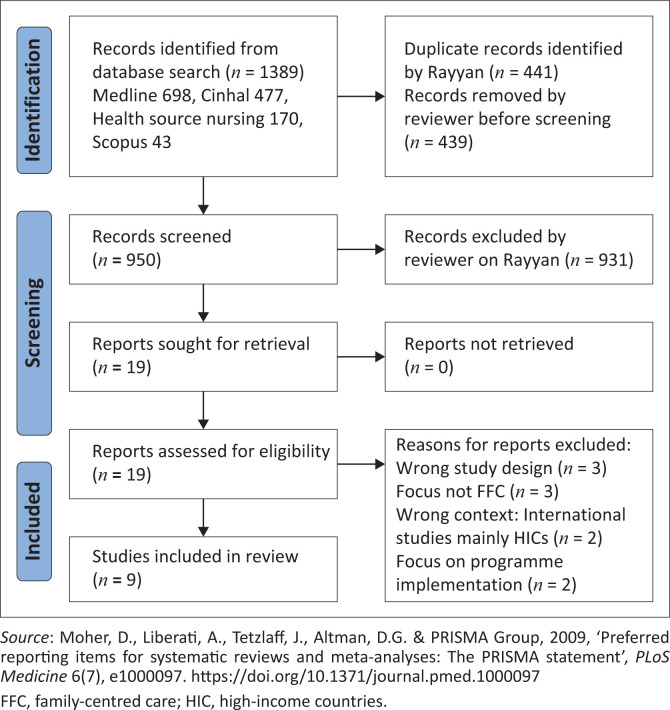
Preferred reporting items for systematic reviews and meta-analyses diagram.

The results of the search are presented in a Preferred reporting items for systematic reviews and meta-analyses (PRISMA) flow diagram (Figure 1). A total of 1389 records were identified from the database search, of which 441 duplicate records were identified by Rayyan, and 439 records were deleted by the reviewer. The 950 remaining records were screened, and 18 studies met the selection criteria in the title and abstract screening phase. Of these, nine were selected for analysis after the full text of the studies was screened.

### Description of the included studies

In all nine studies, the phenomenon of interest was clearly stated, and a suitable qualitative methodology was used to investigate the phenomenon. The data collection methods included interviews, focus groups, observations and field notes. One study included only nurses as participants; the other eight studies included nurses as well as other healthcare practitioners and parents as participants. As noted previously, only data pertaining specifically to nurses were extracted and included in this review. The number of nurses in the studies ranged between 7 and 34 nurses, with one of the studies not specifying the number of nurse participants. The studies were conducted in Rwanda, Ghana, Thailand, Mexico and Iran. Although the two studies from Ghana may have used the same dataset, both studies were included because they report different aspects of the findings and thus provide complementary insights that are relevant to this review. While multiple studies originate from Iran, this reflects a genuine gap in the literature from other LMICs rather than a selection bias. In addition to meeting the inclusion criteria, the Iranian studies also provide perspectives relevant to culturally diverse contexts. Seven studies used thematic analysis, and in two studies, content analysis was employed, with the findings presented as themes. The voices of the participants were adequately represented in the studies. Ethical approval was obtained in all the studies. Methodological limitations include the fact that only four studies included a statement locating the researcher culturally or theoretically, three of the studies noted the possible influence of the researcher on the research, and none of the studies stated their philosophical perspective. Table 1 summarises pertinent aspects of the data extracted from each study, as well as linking the studies to the identified themes.

**TABLE 1:** A summary of records included in the review.

Author (Year)	Phenomenon of interest	Number of nurses	Country	Outcomes	Themes linked to findings
Abukari et al. (2022)	To present the experiences and contextual practices of FCC from the perspectives of families and clinicians in the Ghanaian NICU	Unspecified	Ghana	(1) Contextual practices: respect and dignity, culture and religion and a multidisciplinary approach (2) Family experiences: emotional stress, lack of information and coping strategies (3) Clinician experiences: support, counselling, education and financial problems	Policies and resources Cultural and contextual variables Support and training
Abukari and Schmollgruber (2024)	To describe FCC practice in Ghanaian NICUs to understand the contextual barriers	33 nurses and midwives	Ghana	(1) Family barriers: family stress and anxiety, inadequate information sharing, education, culture and religion (2) Facility barriers: inadequate space and logistics to admit more babies and accommodate families, workload and inadequate staff, restricted entry, negative staff attitudes towards families	Policies and resources Cultural and contextual variables Nurse-family dynamics
Hassankhani et al. (2020)	To explore mothers’ and nurses’ experiences of trust in one another around the caregiving of the hospitalised infant in intensive neonatal care unit	16 nurses	Iran	(1) Gradual and fragile trust of mother-to-nurse (2) Gradual and fragile trust of the nurse-to-mother	Nurse-family dynamics Staff dynamics
Heidari and Mardani-Hamooleh (2020)	To understand the nurses’ perception of FCC in NICUs	18 nurses	Iran	(1) Prerequisite for Providing FCC: suitable facilities, adequate personnel (2) Parents’ Participation: Parents–Neonate’s Attachment, Parents’ Training	Policies and resources Cultural and contextual variables Support and training
Jafari et al. (2023)	To investigate the communication barriers related to personnel and parents for implementing the FCC	1 head nurse 1 matron 14 nurses	Iran	(1) Unprofessional relationships of personnel (2) Mutually ineffective relationships between personnel and parents	Policies and resources Cultural and contextual variables Nurses’ roles and responsibilities Nurse-family dynamics Staff dynamics Support and training
Mendizabal-Espinosa and Warren (2020)	To identify barriers that might explain why healthcare staff struggle to implement infant- and family-centred developmental care programmes	34 nurses	Mexican Republic	(1) The risk of infection from the healthcare professionals’ perspective (2) Risk of infection from the parents’ perspective (3) Infection control and the hierarchy of cleanliness	Policies and resources Cultural and contextual variables Nurse-family dynamics
Mirlashari et al. (2020)	To investigate the perspectives on the challenges of implementing the FCC in the NICU	25 nurses	Iran	(1) Power imbalance: belief in medical authority of health professions, unquestioned physicians̛ power (2) Psychosocial issues: fear, high stress atmosphere, unresolved family difficulties and discouragement of fathers’ involvement in care (3) Structural limitations: policy restriction, organisational limitations	Policies and resources Nurse-family dynamics Staff dynamics
Ndiaye (2020)	To determine parents’ experiences on a neonatal unit in a low-income country, how they and staff perceive the role of parents and if parents’ role as primary carers could be extended.	7 nurses	Rwanda	(1) Inadequate nurse staffing (2) Role of nurses (3) Potential extension of parent’s role (4) Concern about care after discharge	Policies and resources Cultural and contextual variables Nurses’ roles and responsibilities Nurse-family dynamics Support and training
Vetcho et al. (2023)	To identify parents’ and interdisciplinary professionals’ perceptions of FCC and to describe the opportunities to improve FCC	8 nurses	Thailand	(1) Recognising and responding to individual families’ different readiness and their rights and values, (2) Working in a parent-interdisciplinary partnership to provide care, (3) Lacking resources and motivation, (4) Understanding of care requirements and providing help/sympathy. Interdisciplinary professionals often viewed parents’ involvement as an obstacle to providing neonatal care.	Policies and resources Cultural and contextual variables Nurses’ roles and responsibilities Nurse-family dynamics Staff dynamics

Note: Please see full reference list of this article: Wepener, C., Olivier, E. & Gerber, B., 2026, ‘Nurses’ perceptions of family-centred care in neonatal intensive care units: Rapid qualitative evidence synthesis of studies in low- and middle-income countries’, *Health SA Gesondheid* 31(0), a3236. https://doi.org/10.4102/hsag.v31i0.3236 for more information.

FCC, family-centred care; NICU, neonatal intensive care unit.

### Review findings

Line-by-line coding of the data generated 153 codes. These codes were grouped and refined into six themes that synthesise the qualitative research on the perceptions of nurses and the important factors for the practice of FCC in NICUs in LMICs. These themes are described below with quotations from the included studies:

#### Policies and resources

Nurses felt that ‘it was hard to provide FCC nursing care in NICU because the design of the healthcare delivery system did not match and was not ready for FCC’ (Vetcho et al. 2023:51) and also stated that the NICU should have guidelines to ensure that the staff understand how to implement policies (Jafari, Kermanshahi & Vanaki 2023:6). They reported that existing hospital regulations that restrict the access of parents and/or families to the NICU hindered participation (Abukari & Schmollgruber 2024:6). Nurses perceived their working environments to be under-resourced and impractical for FCC, as there was limited space relative to the number of infants needing care, stating that ‘there is no place here’ for the families (Heidari & Mardani-Hamooleh 2020:18). Hospitals with NICU care served large geographical areas; however, certain hospitals had no or limited accommodation for the parents.

#### Cultural and contextual variables

An important theme was neonatal nurses considering differences in a family’s background and needs and how these may impact healthcare decisions and participation. Working in multicultural contexts, as well as in contexts where traditions are shifting, nurses reported a need to be aware of and respect cultural and religious practices, such as wanting the infant to be discharged for a naming ritual, the father needing to be present for making decisions as head of the family or having religious symbols in the NICU. Nurses were aware of the reality of socio-economic differences and how parents had many roles and other responsibilities besides the care of the infant in the NICU:

[*B*]ecause they are different in the level of education, social, and living problems, … these differences influence their readiness to participate. Moreover, parents’ readiness in providing neonatal care depends on parents’ maturity and other factors. (Vetcho et al. 2023:50)

#### Nurses’ roles and responsibilities

The lack of guidelines impacted the nurses’ perceptions of their role and responsibilities towards the families, and a lack of motivation to change was reported in the selected studies. The nurses felt that the infant’s well-being was their core responsibility in the NICU as ‘nurses are there from the first minute to the last minute, the first person who is important for babies to be alive’ (Ndiaye et al. 2020:3). They therefore saw their main priority as caring for the infants and focusing on preventative and promotive care, as well as the prevention of complications. In studies from Rwanda, Iran and the Mexican Republic, the presence of families was perceived to increase infection risk, as illustrated by a Mexican nurse who stated that:

[*T*]he socioeconomic status our population belongs to and their low levels of study make it difficult to inform and help them conduct themselves carefully in the NICU, which evidently increases the risk of infection. (Mendizabal-Espinosa & Warren 2020:316)

Nurses mistrusted the traditional child-rearing practices of parents and regarded it to be ‘the role of the nurse to educate the mother how to take care of the baby’ in the unit (Ndiaye et al. 2020:3), as well as after discharge to prevent re-admission. Additional responsibilities were regarded as increasing their already heavy workload, as observed in this quotation ‘when there are few nurses, we cannot spend time with the parents and listen to what they say about their children’ (Heidari & Mardani-Hamooleh 2020:18). This workload included significant amounts of paperwork. The nurses’ multiple roles and responsibilities left them feeling overwhelmed, frustrated and exhausted.

#### Nurse-family dynamics

Nurses differed in their perceptions about parents’ and families’ involvement in the NICU. Some nurses reported that collaboration with the parents was beneficial for the infants and reassuring for the parents (Abukari, Acheampong & Aziato 2022:4; Heidari & Mardani-Hamooleh 2020:18; Mendizabal-Espinosa 2020:316; Ndiaye et al. 2020:3). Other nurses thought that it was impractical and undesirable to have parents in the NICU (Jafari et al. 2023:6; Mirlashari et al. 2020:e93; Vetcho et al. 2023:51), stating that ‘the parent’s involvement in neonatal care is the obstacle for nursing care. When parents participated in care, they would complicate the situation and induce workload for us’ (Vetcho et al. 2023:51). Nurses perceived some parents’ behaviour to be disrespectful, judgemental and demanding and commented that parents misinformed each other. Nurses from Iran stated that the presence of fathers in the NICU was inappropriate as mothers were feeding their infants, and that the nurses themselves:

[*D*]on’t feel comfortable whenever fathers stay in the unit for a long time. Because some of them pay attention to us rather than their baby! And this situation violates our privacy. (Mirlashari et al. 2020:94)

The communication between the nurses and parents was reported to be challenging because of language, literacy and cultural barriers. Nonetheless, nurses stated that their relationship with the families was important, and that well-timed information communicated in a respectful manner helped to establish trust. In one of the studies, nurses indicated that ‘parents prefer to place decision making power in the hands of hospital staff’ (Mirlashari et al. 2020:93). Nurses reported that they observed how capable parents looked and then decided about their involvement. The infant’s medical condition was noted to impact the level at which nurses allowed the family to be involved in the care of the infant.

#### Staff dynamic

The dynamic between the staff was also perceived to be complicated. The NICU was reported to have a hierarchical system with most managerial and policymaking positions held by doctors (Mirlashari et al. 2020:e94). Nurses felt that the families and other staff members treated them as having a lower rank compared to the doctors. Nurses perceived a negative display of power from their superiors, as shown in the quotation:

I do not accept the behavior of the head nurse. Sometimes, she came into the crowd among all these people and families and behaved so insultingly that I could not raise my head; I cried for an hour. (Jafari et al. 2023:5)

The way the doctors spoke to parents about the nursing staff was also viewed negatively. In addition, nurses reported jealousy among one another as parents would ask for specific nurses to attend to their infant. All the above-mentioned staff tensions were exasperated by serious nursing staff shortages: ‘One major barrier is the inadequate staff to integrate the family into our care in the NICU’ (Abukari & Schmollgruber 2024:7).

#### Support and training

Nurses reported mothers feeling isolated and said that mothers were missing the support they would have received in their communities or had reduced support because ‘unfortunately, cultural transition of our society has made the role of grandparents less visible’ (Heidari & Mardani-Hamooleh 2020:18). This lack of support can lead to families asking for the premature discharge of the infant (Abukari et al. 2022:5). Nurses reported that families needed financial aid and emotional support and that they would benefit from counselling. Nurses also perceived a need for nursing support in the form of emotional support and adequate training in FFC principles and implementation.

## Discussion

This review synthesised evidence on nurses’ perception of FFC in the NICU and identified factors that are important to consider in LMICs. The findings resonate well with previous review findings and highlight contextual constraints and interpersonal challenges faced by nurses in NICUs.

This review found that nurses perceived a lack of policies and resources for the implementation of FFC. Nurses stated that the NICU operates within a hierarchical hospital context and expressed a need for clearly defined policies and implementation guidelines so that they can understand their roles and responsibilities towards the families. Davidson et al. (2017) provide clinical guidelines with evidence-based strategies to optimise the support of the family in intensive care units, which may serve as a reference for hospital policies. The findings of this review show that nurses across the included countries view their primary role as providing medical care of the infant, and that many report lacking the resources to take on additional duties, such as family support, because of understaffing and limited space. Sidek, Marup and Zolkefli (2021) similarly discuss the challenges of balancing family support and other nursing duties and that there is a need for additional support for families in NICUs.

Nurses working in LMIC NICUs also highlight cultural and contextual factors specific to the environment in which the care is provided. It is important to tailor FCC to local contexts (Lazzerini et al. 2024) and to integrate cultural considerations into healthcare delivery in the NICU, particularly those unique to the communities that healthcare professionals serve (Nyaloko et al. 2023). In multicultural contexts, diverse cultural needs are often poorly understood, and it is essential to recognise culturally distinct practices (Carew, Redley & Bloomer 2024). An example is acknowledging the role of grandmothers in the care of infants in non-western collectivist cultures (Aubel 2021). The NICU staff guidelines would thus need to have a measure of flexibility in multicultural contexts, and nurses may need cultural sensitivity training to know how to interact with families from different backgrounds. Lechner, Kukora and Hawes (2024) propose using an updated FCC framework that integrates culturally humble care into the NICU FCC framework.

The dynamics between neonatal nurses and families is another important factor to consider. The current review found that nurses were aware of the benefits of including the mothers in the care of their infants, but they differed in their views on whether this was practical in the NICU. The NICU nurses reported that the mothers of hospitalised infants had other roles and responsibilities, and nurses from the included countries questioned the capabilities of certain mothers to care for their hospitalised infants. The findings of the current review showed that nurses also differed in their view of the role of fathers in the NICU. This ranged from viewing the father’s role as essential in decision-making in Ghana to considering their presence inappropriate in Iranian NICUs. Further research is needed across different contexts to establish how nursing staff and fathers relate, especially given that an integrative review by Merritt (2021), which includes several studies from LMICs, reports that fathers wish to be a part of their infant’s care in the NICU and to develop relationships with the NICU staff. The findings of this review also indicate that the role of the extended family in supporting the parents of the hospitalised infant is an important factor to consider, aligning with Flacking et al. (2022) who discuss the advantages and disadvantages of including ‘important others’ in the NICU. It is thus vital to consider staff-family dynamics to ensure that these do not add to the stress levels in the NICU. To achieve this, the current study emphasises the need for improved communication between nurses and families and the importance of trust building.

Teamwork among healthcare practitioners in the NICU is also important and the current review highlights the need for improved staff dynamics. Oldland et al. (2020) state that positive interpersonal behaviours are one of the domains in the framework of nurses’ responsibilities for quality healthcare, and open effective communication is considered a key element for the success of FCC (Gilstrap 2021; Uema et al. 2020).

According to the nurses included in the reviewed studies, there is a need for greater support for the families of hospitalised infants and for nursing staff themselves, consistent with the findings of Franck et al. (2021). They also reported that additional FCC training would be beneficial. It is not surprising that a study by Kutahyalioglu et al. (2023) has the title: ‘It Takes a Village’ to Implement FCC in the NICU.

### Limitations

The identified studies were conducted in five different LMICs, and the findings are thus not representative of all LMICs. This review focussed on the most current findings, and the COVID-19 pandemic may have limited the number of publications on the topic in this time frame. Another possible limitation is that the review does not include grey literature because of the rapid nature of the review; however, grey literature can be included in future qualitative evidence syntheses (Glenton et al. 2023).

## Conclusion

Evidence from the included studies suggests that the implementation of FCC in NICUs in LMICs is challenging and that specific factors are important to consider when implementing FCC in these contexts. The findings of this study show that the NICUs in LMICs are under-staffed, and although nurses are positive towards the concepts of FCC, they reported a need for clear implementation guidelines within their work settings concerning their roles and responsibilities towards the neonates’ families. Nurses also had a need for training in FCC and for additional counselling support for themselves and the neonates’ families in the stressful and under-resourced NICU environment. Culturally sensitive communication and interactions were considered a vital factor in the success of FCC in NICUs in LMICs.
